# BAP1-Altered Malignant Pleural Mesothelioma: Outcomes With Chemotherapy, Immune Check-Point Inhibitors and Poly(ADP-Ribose) Polymerase Inhibitors

**DOI:** 10.3389/fonc.2021.603223

**Published:** 2021-03-10

**Authors:** Elizabeth Dudnik, Jair Bar, Assaf Moore, Teodor Gottfried, Mor Moskovitz, Julia Dudnik, Tzippy Shochat, Aaron M. Allen, Alona Zer, Ofer Rotem, Nir Peled, Damien Urban

**Affiliations:** ^1^ Thoracic Cancer Service, Davidoff Cancer Center, Rabin Medical Center, Petah Tikva, Israel; ^2^ Sackler Faculty of Medicine, Tel Aviv University, Tel Aviv, Israel; ^3^ Thoracic Oncology Service, Institute of Oncology, Sheba Medical Center, Ramat Gan, Israel; ^4^ Thoracic Cancer Service, Rambam Health Care Campus, Haifa, Israel; ^5^ The Legacy Heritage Oncology Center, Soroka Medical Center, Beersheba, Israel; ^6^ Statistical Consulting Unit, Rabin Medical Center, Petah Tikva, Israel; ^7^ Ben Gurion University of Negev, Beersheba, Israel

**Keywords:** mesothelioma, BAP1, immune check-point inhibitors, PARP inhibitors, chemotherapy

## Abstract

**Objectives:**

Little is known regarding the outcomes of systemic treatments in BAP1-altered malignant pleural mesothelioma (MPM).

**Materials and Methods:**

Forty five patients with MPM [group A: eight MPM patients with BAP1 inactivating mutation/copy number loss (FoundationOne^®^ CDx/TEMPUSxT), selected from the electronic databases of four Israeli cancer centers (ICC); group B: 37 consecutive (years 2016–2018) MPM patients selected from the electronic databases of two ICC—of those six patients without a BAP1 alteration (group B1) and 31 patients not tested for BAP1 (group B2)] were analyzed for ORR, PFS (mRECIST), and OS with 1^st^-line platinum/pemetrexed+/−antiangiogenic drug (CT, n-28), immune check-point inhibitors (ICPi, n-16) and poly (ADP-ribose) polymerase inhibitors (PARPi, n-4). OS since diagnosis (OSDx) was assessed.

**Results:**

There were no differences in ORR or mPFS with CT between the groups: ORR-50% *vs*. 47% *vs*. 50% *vs*. 47% (p>0.9), mPFS-9.1mo (95% CI, 1.2–16.1) *vs*. 9.2mo (95% CI, 2.9–13.3) *vs*. 7.2mo (95% CI, 2.3-NR) *vs*. 10.9mo (95% CI, 2.9–20.3) (p>0.8) in groups A, B, B1, and B2, respectively. There were no differences in ORR or mPFS with ICPi between the groups: ORR-0% *vs*. 27% *vs*. 33% *vs*. 25% (p>0.2), mPFS-2.5mo (95% CI, 1.4–3.7) *vs*. 3.0mo (95% CI, 1.3–10.5) *vs*. 2.0mo (95% CI, 1.9-NR) *vs*. 4.5mo (95% CI, 0.3–10.5) (p>0.3) in groups A, B, B1, and B2, respectively. In group A, no responses were seen with PARPi; mPFS with PARPi was 1.8mo (95% CI, 1.8-NR). OSDx was 98.3mo (95% CI, 9.7–98.3) *vs*. 19.4mo (95% CI, 9.7–47.3) *vs*. 18.8mo (95% CI, 8.5-NR) *vs*. 19.5mo (95% CI, 8.3–82.2) in groups A, B, B1, and B2, respectively (p>0.3).

**Conclusions:**

BAP1-altered MPM, as compared to non-selected MPM, is characterized by similar efficacy of CT and ICPi. Numerically longer OS in BAP1-altered MPM may reflect favorable tumor biology. No responses were observed with PARPi.

## Introduction

Mesothelioma is a rare malignancy that originates from mesothelial cells, mostly from the pleura [malignant pleural mesothelioma (MPM)], with an incidence rate of 1.4 cases per 100,000 population in the United States (1, 2). The median OS (mOS) of patients with MPM is in the range of 5.9–12.6 months, 1-year survival is 21–55%, and 5-year survival is about 10% (2–5).

The recommended 1^st^-line regimen for advanced MPM is a combination of cisplatin and pemetrexed (6). The addition of bevacizumab to cisplatin and pemetrexed results in a moderate survival improvement (7), and it is recommended for use in selected patients (8). Carboplatin-pemetrexed is also appropriate when cisplatin is contraindicated (8–12). This therapy is associated with an objective response rate (ORR) of 34–41%, median progression-free survival (mPFS) of 5.7–9.2 months, and mOS of 12.1–18.8 months (6, 7).

Immune checkpoint inhibitors (ICPi) in MPM were initially assessed after progression on platinum/pemetrexed. In this clinical scenario, therapy with programmed cell death-1 (PD-1) inhibitors or programmed cell death ligand-1 (PD-L1) inhibitors is associated with an ORR of 9–29%, mPFS of 2.6–4.1 months, and mOS of 11.5–12 months (13–17). Numerically better results have been demonstrated with the combination of a PD-1/PD-L1 inhibitor with a cytotoxic T-lymphocyte-associated protein 4 (CTLA-4) inhibitor (ORR of 25%, mPFS of 5.6–5.7 months, mOS of 16–16.6 months) (18, 19). Recently, the combination of nivolumab, a PD-1-inhibitor, with ipilimumab, a CTLA-4 inhibitor, in treatment-naïve MPM, resulted in better OS as compared to therapy with platinum/pemetrexed (20).

BRCA1 associated protein-1 (BAP1) is responsible for de-ubiquitination of histones and, as a result, protein transcription and cell cycle regulation (21), it also acts as a homologous recombination deoxyribonucleic acid (DNA) repair component found in the BRCA1/BARD1 complex (22). Pathogenic germline variants of BAP1 are associated with various malignancies, including mesothelioma (23–25); 1–7% of malignant mesotheliomas are attributable to a germline mutation in BAP1 (26–28). Additionally, between 20 and 64% of MPM harbor somatic inactivating aberrations in BAP1, including point mutations, copy number loss, and rearrangements (22, 26, 29–38). In MPM, the presence of either germline or somatic BAP1 aberration has been associated with prolonged OS in most of the genomic analyses performed (23, 27, 31, 34–37, 39, 40).

Little is known about the value of BAP1 alterations as potential predictive biomarkers and as targets for various systemic treatments in MPM. It has been hypothesized that BAP1-altered MPM are characterized by extremely high sensitivity to platinum-based chemotherapy—similarly to BRCA2-mutant ovarian cancer (41). This hypothesis was based on the assumption that BAP1-altered MPM cells cannot efficiently repair platinum-induced DNA cross-links. It has also been suggested that BAP1-altered MPM might be susceptible to poly (ADP-ribose) polymerase inhibitors (PARPi) (22, 42, 43). Mesotheliomas harboring BAP1 aberrations are characterized by elevated immune signaling and inflammatory tumor microenvironment (36, 44), and, therefore, may predict long-term responses with ICPi.

However, llittle is known regarding the clinical outcomes of the above-mentioned systemic treatments in BAP1-mutant MPM. We conducted this retrospective analysis, aiming to compare the clinical outcomes with platinum/pemetrexed+/−antiangiogenic drugs, ICPi and PARPi in patients with BAP1-altered MPM to outcomes in BAP1-wild type or non-selected MPM patients.

## Materials and Methods

### Patient Selection and Group Assignment

Forty-three consecutive patients with histologically confirmed MPM diagnosed in January 2016–December 2018 were identified through electronic databases of two Israeli cancer centers (Davidoff Cancer Center, Rabin Medical Center, Beilinson Campus and Institute of Oncology, Sheba Medical Center, Tel HaShomer). Of the selected patients, 12 patients (28%) underwent next-generation sequencing of their tumors, and BAP1 tumor status has been determined. The selected patients were divided into group A (n-6; tumors with a BAP1 inactivating mutation/copy number loss) [BAP1 status was determined using FoundationOne^®^ CDx (n-5) (45), or TEMPUSxT (n-1) (46)], and group B (n-37; tumors without a BAP1 alteration or tumors not tested for the presence of a BAP1 alteration). Patients in group B were further divided into group B1 (n-6; tumors without a BAP1 alteration) [BAP1 status was determined using FoundationOne^®^ CDx (n-3) (45), FoundationOne^®^ Liquid (n-1) (47), TEMPUSxT (n-1) (46), and GPS Cancer™ (n-1) (48)], and group B2 (n-31; tumors not tested for the presence of a BAP1 alteration). A search aiming at identifying additional patients with a BAP1-mutant MPM was performed within the Israeli Lung Cancer Group, and two additional patients meeting the above-mentioned criteria for inclusion in group A were identified [BAP1 mutation was diagnosed by FoundationOne^®^ CDx in both cases (45)]. After adding these 2 patients, the study cohort reached 45 patients overall, of those: 8 patients in group A and 37 patients in group B (6 patients in group B1 and 31 patients in group B2).

### Study Design and Assessments

Baseline demographic (including asbestos exposure and family history of MPM), clinical [including European Organization for Research and Treatment of Cancer (EORTC) prognostic score (3), Cancer and Leukemia Group B

(CALGB) prognostic score (4, 5), surgery and radiotherapy type, systemic treatment type] and pathologic characteristics [including histological type, PD-L1 expression, tumor mutational burden (TMB), MSI status/mismatch repair status (MMR)] were collected and compared between the groups.

PD-L1 assessment was done by immunohistochemistry (IHC) using 22C3 PharmDx antibody on either Dako 22C3 PD-L1 IHC platform (Dako, Carpinteria, CA) or Ventana’s BenchMark XT platform (Ventana Medical Systems, Tucson, AZ) (49, 50). PD-L1 tumor proportion score (TPS), which is the percentage of tumor cells showing partial or complete membrane staining, was determined and classified as negative, intermediate, or high (TPS of <1%, 1–49%, and >=50%, respectively) (49). TMB was calculated according to either the FoundationOne^®^ CDx algorithm (45, 47), TEMPUSxT algorithm (46), or GPS Cancer™ algorithm (48)—according to the next-generation sequencing platform used in each case. MSI status was determined using either the FoundationOne^®^ CDx algorithm (45, 47), or TEMPUSxT algorithm (46), and reported as “MSI-high” and “MSI-stable.” MMR status was assessed by IHC staining for MLH-1 (mouse, clone M1 Ventana), MSH-2 (mouse, G-219-1129 Ventana), MSH-6 (mouse, clone 44 Ventana or SP93 Cell Marque), and PMS-2 (rabbit, EPR 3947 Ventana) proteins on Ventana’s BenchMark XT platform (Ventana Medical Systems, Tucson, AZ), and reported as “MMR deficient” or “MMR proficient.”

ORR, PFS, and OS were assessed and compared for each different systemic treatment modality administered (platinum/pemetrexed chemotherapy, ICPi). For that purpose, only therapies administered for the treatment of advanced-stage disease were considered (neo-adjuvant/adjuvant systemic therapies were excluded from the analysis); for platinum-based combinations only 1^st^ line treatments were analyzed (re-challenge with platinum-based combination were not included in the comparison). Additionally, OS since disease diagnosis (OSDx) was compared between the groups. In group A, ORR, PFS, and safety with PARPi were assessed. Univariate analyses of PFS and OS with platinum/pemetrexed+/−antiangiogenic drug, ICPi, and OSDx were performed using Cox proportional-hazards regression model.

ORR and PFS were assessed using Modified Revised Response Evaluation Criteria in Solid Tumors (mRECIST) used for response assessment in MPM (51); radiological assessment was done by the investigators. PFS was calculated from treatment initiation until disease progression or death; the outcome was censored if a patient was alive without known progression of the disease at the time of last follow-up. OS was calculated from the day of treatment initiation until death; the outcome was censored if a patient was alive at the time of last follow-up. OSDx was calculated similarly, since MPM diagnosis. Adverse events were graded using Common Terminology Criteria for Adverse Events, version 4.03 (CTCAE, v. 4.03) (52).

The study was conducted in accordance with the principles of good clinical practice, and institutional review board approval was obtained before the study initiation.

### Statistical Analysis

The sample size was determined by the available patients meeting the inclusion criteria. The statistical analysis was generated using SAS Software, version 9.4 (53).

Categorical variables were presented by numbers and percentiles, medians and ranges were reported for continuous variables. Fisher’s exact test and T-test were used to compare the baseline demographic, clinical, and pathologic characteristics. PFS and OS were assessed by the Kaplan-Meier method, with the log-rank test for the comparison. The Cox proportional-hazards regression model was used for univariate and multivariate OS analysis. All reported p-values are based on two-sided hypothesis tests.

## Results

### Patient and Tumor Characteristics

Baseline demographic and clinical characteristics of patients are presented in **Table 1**. The vast majority of patients were mid-aged males, tumors with epithelioid histology predominated; the majority of tumors were “good-prognosis” by EORTC and CALGB prognostic scoring systems. Patients in group A were younger as compared to patients in group B1 (p-0.02). Patents in group A were more likely to have a family history of malignancy (including breast cancer, gastric cancer, cancer of ovary, lung cancer, colorectal cancer, and CNS tumors), and less likely to have an asbestos exposure—these differences were not statistically significant. Interestingly, 1 patient appearing to have no BAP1 alteration in his tumor (using GPS Cancer™) had a family history of MPM in two brothers. More patients in group A, as opposed to group B, received platinum-based chemotherapy as a 1^st^-line treatment for advanced disease (p-0.02); as expected, only patients in group A received PARPi. No other imbalances in terms of clinical baseline and treatment characteristics between the groups were observed.

**Table 1:** Baseline and treatment characteristics of patients with malignant pleural mesothelioma in Group A (BAP1 inactivating mutation/copy number loss, n-8) and Group B (without a BAP1 alteration/not tested, n-37).

	Group A (n-8)	Group B (n-37)	Group B1 (n-6)	Group B2 (n-31)	p value (between A and B)	p value (between A and B1)
**Age at diagnosis, years ** **(median, range)**	65 (25-76)	69 (30-93)	77 (71-90)	66 (30-93)	0.2	0.02
**Sex, n (%)**					0.7	0.6
**Female**	3 (37)	11(30)	1 (17)	10 (32)		
**Male**	5 (63)	26 (70)	5 (83)	21 (68)		
**Asbestos exposure, n (%)**					0.4	1.0
**Yes**	2 (25)	15 (41)	2 (33)	13 (42)		
**No**	5 (63)	15 (41)	4 (67)	11 (35)		
**NA**	1 (12)	7(18)	0 (0)	7 (23)		
**Family history of malignancy, n (%)**	2 (25)	4 (11)	1 (17)	3 (10)	1.0	1.0
**Histology, n (%)**					0.6	1.0
**Epithelioid**	7 (88)	31 (85)	6 (100)	25 (82)		
**Sarcomatoid **	0 (0)	2 (5)	0 (0)	2 (6)		
**Biphasic**	1 (12)	2 (5)	0 (0)	2 (6)		
**NA**	0 (0)	2 (5)	0 (0)	2 (6)		
**PD-L1 TPS, n (%)**					0.03	0.08
**>50%**	2 (25)	0 (0)	0 (0)	0 (0)		
**1-50%**	0 (0)	5 (14)	3 (50)	2 (6)		
**<1%**	1 (12)	2 (5)	1 (17)	1 (3)		
**NA**	5 (63)	30 (81)	2 (33)	28 (91)		
**MSI high/MMR deficient, n (%)**	0 (0)*	0 (0)**	0 (0)	0 (0)	0.4	0.6
**TMB, mut/Mb, (median, range)**	3 (0.8-5)¥	1.5 (0-2)¥¥	1.5 (0-2)¥¥	NA	0.2	0.2
**Stage at diagnosis (AJCC Cancer** **Staging, 8^th^ edition), n (%)**					0.5	0.5
**I/II**	1 (12)	9 (24)	2 (33)	7 (22.5)		
**III**	3 (38)	19 (51)	3 (50)	16 (52)		
**IV**	3 (38)	8 (22)	1 (17)	7 (22.5)		
**NA**	1 (12)	1 (3)	0 (0)	1 (3)		
**ECOG PS at diagnosis, n (%)**					1.0	1.0
**0/1**	7 (88)	30 (81)	6 (100)	24 (78)		
**2/3/4**	1 (12)	5 (14)	0 (0)	5 (16)		
**NA**	0 (0)	2 (5)	0 (0)	2 (6)		
**EORTC prognostic scoring system**					0.6	1.0
**Good-prognosis**	5 (63)	22 (59)	5 (83)	17 (55)		
**Poor-prognosis**	1 (12)	11 (30)	0 (0)	11 (35)		
**NA**	2 (25)	4 (11)	1 (17)	3 (10)		
**CALGB prognostic scoring system**					0.7	0.5
**1/2**	2 (25)	12 (32)	3 (50)	9 (29)		
**3/4**	2 (25)	18 (49)	2 (33)	16 (51)		
**5/6**	1 (12)	3 (8)	0 (0)	3 (10)		
**NA**	3 (38)	4 (11)	1 (17)	3 (10)		
**Surgery, n (%)**					1.0	1.0
**EPP**	1 (12)	6 (16)	1 (17)	5 (16)		
**Decortication**	1 (12)	1 (3)	0 (0)	1 (3)		
**Pleurodesis**	1 (12)	7 (18)	3 (50)	4 (13)		
**Chest radiotherapy, n (%)**					0.4	1.0
**Definitive **	2 (25)	9 (24)	1 (17)	8 (26)		
**Palliative**	3 (38)	7 (18)	2 (33)	5 (16)		
**Platinum/pemetrexed+/-antiangiogenic agents (as a 1^st^-line treatment for advanced-stage disease), n (%)**	8 (100)	20 (54)	4 (67)	16 (52)	0.02	0.2
**ICPi, n (%)**	3 (38)	13 (35)	4 (67)	9 (29)	1.0	0.6
**PARPi, n (%)**	4 (50)	0 (0)	0 (0)	0 (0)	0.0005	0.0005

AJCC, American Joint Committee on Cancer; CALGB, Cancer and Leukemia Group B; BAP1, BRCA1 associated protein-1; ECOG PS, Eastern Cooperative Oncology Group performance status score; EORTC, European Organization for Research and Treatment of Cancer; EPP, extra-pleural pneumonectomy; ICPi, immune check-point inhibitors; MMR, mismatch repair; MSI, microsatellite instability; mut/Mb, mutations per megabase; NA, not available/not applicable; PARPi, poly (ADP-ribose) polymerase inhibitors; PD-L1, programmeddeath ligand 1; TMB, tumor mutational burden; TPS, tumor proportion score.*n-8; **n-6; ¥n-5; ¥¥n-4.

From the molecular perspective, the proportion of tumors with TPS ≥50% was higher in group A as opposed to group B (25 *vs*. 0%, p-0.03); no significant differences in terms of tumor PD-L1 expression between groups A and B1 were observed. None of the 14 tumors tested (8 in group A, and 6 in group B) had an MSI-high or MMR-deficient status. Median TMB was low in either group and comprised three mutations per megabase (mut/Mb) (range, 0.8–5), 1.5 mut/Mb (range, 0–2), and 1.5 mut/Mb (range, 0–2) in 5, 4, and 4 tumors tested in groups A, B, and B1, respectively, without significant differences between the groups (**Table 1**). The BAP1 alterations diagnosed in patients in group A (all predicted to be associated with loss of BAP1 function(were as follows: BAP1 Q684 mutation, BAP1 F660fs*32 mutation, BAP1 loss exons 9-17 mutation, BAP1 E198fs*45 mutation, BAP1 splice site 375 + 2T>C mutation, BAP1 loss exons 13-17, BAP1 loss, BAP1 copy number loss. The co-existing alterations observed in BAP1-altered tumors are listed in **Table 2**. No matched normal DNA was available for the analysis. Upon our request, in seven out of eight cases included in group A, an additional review of the molecular data has been done by Foundation Medicine, however, no differentiation could be made with regards to somatic or germline nature of the BAP1 alterations (**Table 2**).

**Table 2:** BAP1 alterations and co-existing genomic alterations, TMB and MSI status in patients with advanced BAP1 - altered MPM.

Patients	BAP1 alteration	Somatic/germline nature of BAP1 alteration	Co-existing genomic alterations	TMB, muts/Mb	MSI
**#1**	BAP1 Q684	NA	CDKN2A/B loss; NOTCH3 R1893	4	MS-stable
**#2**	BAP1 F660fs*32	NA The sample failed the copy BAP1 number quality metrics for SGZ calling, whereas somatic/germline calling was still unavailable with manual review	MLL2 splice site 14382+1G>T	NA	NA
**#3**	BAP1 loss exons 9-17	NA Somatic/germline calling is not available for copy number alteration events	NF2 W74; CDKN2A p16INK4a R80* and p14ARF P94L; CREBBP complex rearrangement	3	MS-stable
**#4**	BAP1 E198fs*45	NA The sample failed the copy number quality metrics for SGZ calling, whereas somatic/germline calling was still unavailable with manual review	DNMT3A D529fs*16	NA	NA
**#5**	BAP1 splice site 375+2T>C	NA	HGF amplification; CDKN2A/B loss; PBRM1 deletion exons 8-12	5	MS-stable
**#6**	BAP1 loss exons 13-17	NA Somatic/germline calling is not available for copy number alteration events	TP53 splice site 9209_993+70del153	1	MS-stable
**#7**	BAP1 loss	NA Somatic/germline calling is not available for copy numberalteration events	KDM6A loss; PBRM1 loss exons 23-30	NA	NA
**#8**	BAP1 copy number loss	NA Somatic/germline calling is not available for copy number stable alteration events	PBRM1 copy number loss	0.8	MS-stable

BAP1, BRCA1 associated protein-1; MSI, microsatellite instability; mut/Mb, mutations per megabase; NA, not determined; TMB, tumor mutational burden.

### ORR, PFS, and OS With Platinum/Pemetrexed+/−Antiangiogenic Agents

Median follow-up since diagnosis of MPM was 18.7 months [interquartile range (IQR), 10.2–31.8], 12.4 months (IQR, 7.8–29.5), 14.7 months (IQR, 8.5–18.8), and 11.2 months (IQR, 7.5–31.2) in groups A, B, B1, and B2, respectively.

Twenty-eight patients [8 patients (100%) in group A, 20 patients (54%) in group B, 4 patients (67%) in group B1, and 16 patients (52%) in group B2] were treated with 1^st^-line platinum/pemetrexed chemotherapy. Of those, nine patients [five patients (62% of receiving platinum/pemetrexed) in group A and four patients (20% of receiving platinum/pemetrexed) in group B (including 1 patient in group B1 and three patients in group B2)] received chemotherapy in combination with bevacizumab. Two additional patients in group B (of those, 1 patient in group B1 and 1 patient in group B2) received 1^st^-line platinum/pemetrexed chemotherapy+/− nintedanib within the LUME-Meso clinical trial (ClinicalTrials.gov Identifier: NCT01907100).

In group A, four patients (50%) had a partial response (PR), three patients (38%) had stable disease (SD), and one patient (12%) had disease progression (PD). In group B, one patient (5%) developed complete response (CR), eight patients (42%) developed PR, seven patients (37%) experienced SD, three patients (16%) had PD, and one patient was not evaluable for response assessment (the treatment was stopped before radiological assessment was done). In group B1, two patients (50%) developed PR, and two patients (50%) experienced SD. In group B2, one patient (7%) developed CR, six patients (40%) developed PR, five patients (33%) experienced SD, three patients (20%) had PD, and one patient was not evaluable for response assessment. ORR with 1^st^-line platinum/pemetrexed chemotherapy+/− antiangiogenic agents comprised 50 and 47% in groups A and B, respectively (p-0.97). In groups A, B1, and B2, ORR was 50, 50, and 47%, respectively (p-1.0).

**Figure 1:**
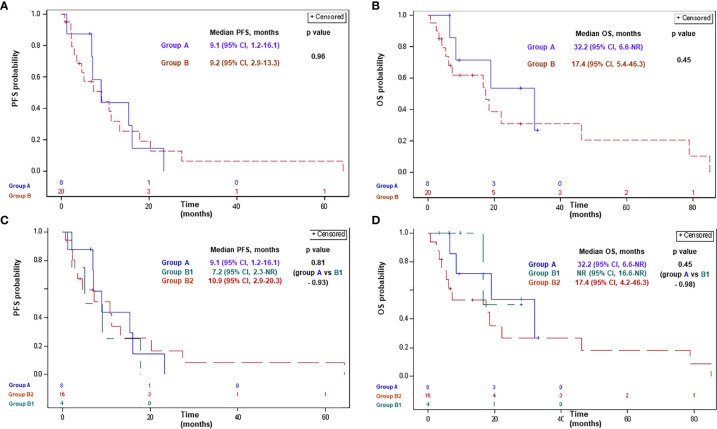
Progression-free survival **(A, C)** and overall survival **(B, D)** with platinum-based chemotherapy in patients with advanced MPM according to BAP1 mutation status. Group A: MPM with a BAP1 inactivating mutation/copy number loss; group B: MPM without a BAP1 alteration/not tested; group B1: MPM without a BAP1 alteration; group B2: MPM not tested for a BAP1 alteration. BAP1, BRCA1 associated protein-1; CI, confidence interval; MPM, malignant pleural mesothelioma; NR, not reached; OS, overall survival; PFS, progression-free survival.

Of patients treated with 1^st^-line platinum/pemetrexed chemotherapy+/− antiangiogenic agents, 7 patients (87%) in group A, 17 patients (85%) in group B, 4 patients (100%) in group B1, and 13 patients (81%) in group B2 had progressed or died. Median PFS comprised 9.1 months [95% confidence interval (CI), 1.2–16.1] and 9.2 months (95% CI, 2.9–13.3) in groups A and B, respectively (p-0.96; **Figure 1**). Median PFS comprised 9.1 months (95% CI, 1.2–16.1), 7.2 months [95% CI, 2.3-not reached (NR)], and 10.9 months (95% CI, 2.9-20.3) in groups A, B1, and B2, respectively (p>0.8 for each comparison; **Figure 1**).

Of patients treated with 1^st^-line platinum/pemetrexed chemotherapy+/− antiangiogenic agents, 4 (50%), 14 (70%), 1 (25%), and 13 (93%) patients had died in groups A, B, B1, and B2, respectively. Median OS with 1^st^-line platinum/pemetrexed chemotherapy+/− antiangiogenic agents comprised 32.2 months (95% CI, 6.6-NR) and 17.4 months (95% CI, 5.4–46.3) in groups A and B, respectively (p-0.45; **Figure 1**). Median OS comprised 32.2 months (95% CI, 6.6-NR), NR (95% CI, 16.6-NR), and 17.4 months (95% CI, 4.2–46.3) in groups A, B1, and B2, respectively (p>0.4 for each comparison; **Figure 1**).

In the univariate analysis, the only variables which correlated with PFS and OS with 1^st^-line platinum/pemetrexed chemotherapy+/− antiangiogenic agents were tumor histology and EORTC risk score (**Table 3**). Presence or absence of BAP1 alteration did not affect PFS or OS with 1^st^-line platinum/pemetrexed chemotherapy+/− antiangiogenic agents in a significant manner.

**Table 3:** Univariate analysis of PFS and OS with platinum/pemetrexed+/antiangiogenic agent (A), ICPi (B) and OS since diagnosis of malignant pleural mesothelioma (C) by the Cox proportional-hazards regression model.

A	PFS				OS			
Parameter	HR	95% HR CI		p value	HR	95% HR CI		p value
Platinum/pemetrexed+/-antiangiogenic agent								
Age	1.01	0.99	1.04	0.25	1.03	0.99	1.07	0.08
Sex (male *vs* female)	2.64	0.88	7.78	0.08	3.63	0.88	14.98	0.07
Histology (sarcomatoid *vs* epithelioid)	13.02	2.01	84.30	0.007	11.22	1.84	68.44	0.008
Histology (biphasic *vs* epithelioid)	12.12	2.02	72.83	0.006	5.62	1.04	30.26	0.04
No BAP1 mutation/not tested vs BAP1 mutation present	0.99	0.40	2.42	0.97	1.44	0.46	4.45	0.53
ECOG PS at chemotherapy initiation (2-4 *vs* 0/1)	10.48	0.65	167.75	0.10	3.41	0.54	21.41	0.19
EORTC prognostic scale (poor risk *vs* good-risk)	8.96	2.01	39.88	0.004	9.33	2.13	40.83	0.003
CALGB prognostic scale (3/4 *vs* 1/2)	1.07	0.39	2.93	0.90	2.08	0.64	6.82	0.23
CALGB prognostic scale (5/6 *vs* 1/2)	1.49	0.33	6.78	0.60	2.34	0.44	12.45	0.32
B		ICPi						
Age	0.97	0.92	1.03	0.37	0.95	0.89	1.03	0.21
Sex (male *vs* female)	0.86	0.22	3.32	0.83	0.96	0.21	4.42	0.96
Histology (biphasic *vs* epithelioid)	12.40	1.17	131.77	0.04	7.14	0.99	51.53	0.05
No BAP1 mutation/not tested vs BAP1 mutation present	0.44	0.09	2.08	0.30	1.07	0.23	4.92	0.93
ECOG PS at ICPi initiation (2-4 *vs* 0/1)	3.56	0.47	27.19	0.22	2.21	0.31	15.93	0.43
EORTC prognostic scale (poor risk *vs* good-risk)	12.96	0.81	207.57	0.07	12.41	0.77	199.35	0.07
CALGB prognostic scale (3/4 *vs* 1/2)	1.92	0.41	9.01	0.41	3.61	0.43	30.23	0.24
CALGB prognostic scale (5/6 *vs* 1/2)	2.63	0.28	25.12	0.40	1.20	0.10	14.51	0.88
C	OS since diagnosis							
Age				1.02	0.99	1.05	0.07	1.02
Sex (male *vs* female)					2.93	1.11	7.75	0.03
Histology (sarcomatoid *vs* epithelioid)					13.98	2.68	73.01	0.002
Histology (biphasic *vs* epithelioid)					3.24	0.79	13.32	0.10
No BAP1 mutation/not tested vs BAP1 mutation present					1.56	0.55	4.41	0.40
ECOG PS at diagnosis (2-4 *vs* 0/1)					3.53	1.11	11.19	0.03
Surgery (EPP/decortication) *vs* none					0.42	0.14	1.19	0.10
Radiotherapy *vs* none					0.77	0.36	1.67	0.51
Platinum-based chemotherapy *vs* none					1.54	0.57	4.16	0.39
ICPi *vs* none					0.80	0.37	1.76	0.58
EORTC prognostic scale (poor risk *vs* good-risk)					2.80	1.19	6.59	0.02
CALGB prognostic scale (3/4 *vs* 1/2)					1.55	0.63	3.83	0.34
CALGB prognostic scale (5/6 *vs* 1/2)					1.91	0.49	7.53	0.35

BAP1, BRCA1 associated protein-1; CALGB, Cancer and Leukemia Group B; CI, confidence interval; ECOG PS, Eastern Cooperative Oncology Group performance status score; EORTC, European Organization for Research and Treatment of Cancer; EPP, extra-pleural pneumonectomy; HR, hazard ratio; ICPi, immune check-point inhibitors; OS, median overall survival; PFS, median progression-free survival.

### ORR, PFS, and OS With ICPi

A total of 16 patients were treated with ICPi: 3 patients (38%) in group A and 13 patients (35%) in group B [including 4 patients (67%) in group B1 and 9 patients (29%) in group B2]. Of these, 12 patients [2 patients (67% of receiving ICPi) in group A and 10 patients (77% of receiving ICPi) in group B] received PD-1/PD-L1 inhibitors, and 4 patients [1 patient [33% of receiving ICPi] in group A and 3 patients (23% of receiving ICPi) in group B] received a combination of a PD-1/PD-L1 inhibitor with another ICPi (mostly, CTLA-4 inhibitor). In group B1, all four patients (100% of receiving ICPi) were treated with PD-1/PD-L1 inhibitors, whereas in group B2, six patients (67% of receiving ICPi) were treated with PD-1/PD-L1 inhibitors, and three additional patients (33% of receiving ICPi) received a combination of a PD-1/PD-L1 inhibitor with another ICPi.

In group A, two patients (100%) had PD, and one patient was not evaluable for response assessment. In group B, three patients (27%) achieved PR, four patients (36.5%) experienced SD, four patients (36.5%) had PD, and two patients were not evaluable for response assessment. In group B1, one patient (33%) achieved PR, two patients (67%) had PD, and one patient was not evaluable for response assessment. In group B2, two patients (25%) achieved PR, four patients (50%) experienced SD, two patients (25%) had PD, and one patient was not evaluable for response assessment. ORR with ICPi comprised 0 and 27% in groups A and B, respectively (p-0.28), and 0, 33, and 25% in groups A, B1, and B2, respectively (p-0.35).

**Figure 2:**
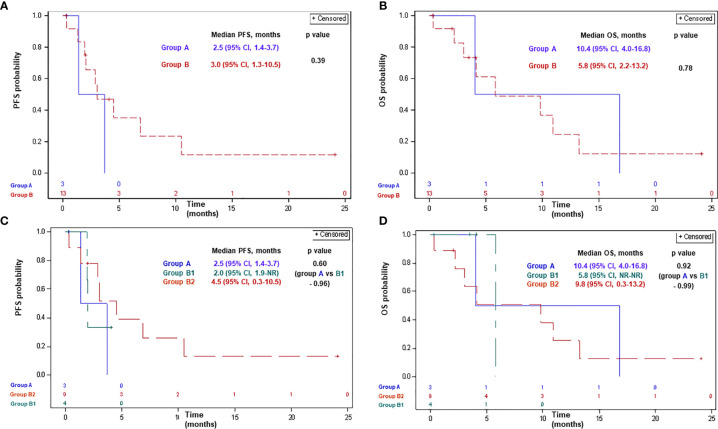
Progression-free survival **(A, C)** and overall survival **(B, D)** with ICPi in patients with advanced MPM according to BAP1 mutation status. Group A: MPM with a BAP1 inactivating mutation/copy number loss; group B: MPM without a BAP1 alteration/not tested; group B1: MPM without a BAP1 alteration; group B2: MPM not tested for a BAP1 alteration. BAP1, BRCA1 associated protein-1; CI, confidence interval; ICPi, immune check-point inhibitors; MPM, malignant pleural mesothelioma; NR, not reached; OS, overall survival; PFS, progression-free survival.

Of patients receiving ICPi, two (67%), nine (69%), two (50%), and seven (78%) patients had progressed or died in groups A, B, B1, and B2, respectively. Median PFS with ICPi comprised 2.5 months (95% CI, 1.4–3.7) and 3.0 months (95% CI, 1.3–10.5) in groups A and B, respectively (p-0.39; **Figure 2**). Median PFS was 2.5 months (95% CI, 1.4–3.7), 2.0 months (95% CI, 1.9-NR), and 4.5 months (95% CI, 0.3–10.5) in groups A, B1, and B2, respectively (p>0.5 for each comparison; **Figure 2**).

Of patients receiving ICPi, 2 (67%), 8 (61%), 1 (25%), and 7 (78%) patients had died in groups A, B, B1, and B2, respectively. Median OS with ICPi comprised 10.4 months (95% CI, 4.0–16.8) and 5.8 months (95% CI, 2.2–13.2) in groups A and B, respectively (p-0.78; **Figure 2**). Median OS was 10.4 months (95% CI, 4.0–16.8), 5.8 months (95% CI, NR-NR), and 9.8 months (95% CI, 0.3–13.2) in groups A, B1, and B2, respectively (p>0.9 for each comparison; **Figure 2**).

In the univariate analysis, the only variable which correlated with PFS and OS with ICPi was tumor histology (**Table 3**). Presence or absence of BAP1 alteration did not affect PFS or OS with ICPi.

### ORR, PFS With PARPi

Four patients in group A were treated with PARPi: two patients received veliparib, two patients received olaparib, and one patient was also treated with the combination of carboplatin and olaparib after olaparib failure. Olaparib was administered at a dose of 300 mg #2/d; veliparib was administered at a dose of 200 mg#2/d; the combination included carboplatin AUC-2 weekly and olaparib 300 mg #2/d. No objective responses with PARPi occurred; three patients had PD, and one patient achieved SD for 3.4+ months (SD is ongoing at the time of the analysis). PFS with PARPi comprised 3.4+, 1.9, 1.8, and 1.8 months; median PFS was 1.8 months (95% CI, 1.8-NR). The patient treated with the combination of carboplatin and olaparib after olaparib failure demonstrated PD 1.5 months after the initiation of the combined treatment.

One patient developed grade 3 thrombocytopenia during olaparib treatment; one patient developed grade 3 fatigue and grade 3 anorexia during veliparib treatment. The combined treatment was not associated with any treatment-related adverse events.

### OS Since MPM Diagnosis

**Figure 3:**
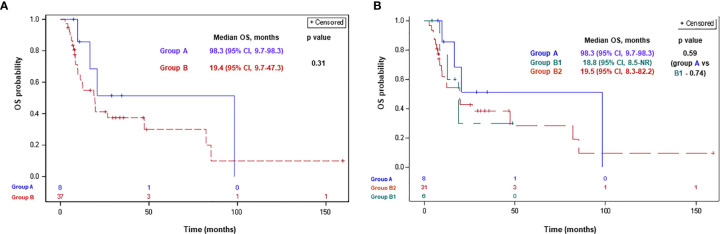
Overall survival since MPM diagnosis according to BAP1 mutation status for groups A, B **(A)** and A, B1, B2 **(B)**. Group A: MPM with a BAP1 inactivating mutation/copy number loss; group B: MPM without a BAP1 alteration/not tested; group B1: MPM without a BAP1 alteration; group B2: MPM not tested for a BAP1 alteration. BAP1, BRCA1 associated protein-1; CI, confidence interval; ICPi, immune check-point inhibitors; MPM, malignant pleural mesothelioma; NR, not reached; OS, overall survival.

Of 45 patients included in the analysis, 4 (50%), 23 patients (62%), 3 (50%), and 20 (64%) patients in groups A, B, B1, and B2, respectively, had died. Median OSDx was 98.3 months (95% CI, 9.7–98.3) and 19.4 months (95% CI, 9.7–47.3) in groups A and B, respectively (p-0.31; **Figure 3**). Median OSDx was 98.3 months (95% CI, 9.7–98.3), 18.8 months (95% CI, 8.5-NR), and 19.5 months (95% CI, 8.3–82.2) in groups A, B1, and B2, respectively (p>0.5 for each comparison; **Figure 3**).

In the univariate analysis, the variables which significantly correlated with OSDx were sex, tumor histology, Eastern Cooperative Oncology Group performance status (ECOG PS) at diagnosis, and EORTC prognostic risk score (**Table 3**). Presence or absence of BAP1 alteration did not demonstrate a statistically significant correlation with OSDx.

## Discussion

To the best of our knowledge, our series represents one of the first one reporting on different systemic treatment outcomes in BAP1-altered MPM patients. According to our observation, 1^st^-line platinum/pemetrexed chemotherapy in this category of patients was associated with an ORR of 50% and mPFS of 9.1 months (95% CI, 1.2–16.1). These results were similar to the results observed in non-selected MPM patients [ORR of 47%, p-0.97 and mPFS of 9.2 months (95% CI, 2.9–13.3), p-0.96]—despite the fact, that higher proportion of patients with BAP1-mutant MPM (62%), as compared to non-selected MPM patients (20%), received the treatment in combination with bevacizumab. Moreover, similar ORR (50%, p-1.0) and mPFS [7.2 months (95% CI, 2.3-NR), p-0.93] were seen in wild-type BAP1 tumors. These results are also in line with the previously reported in the literature with platinum/pemetrexed-bevacizumab in non-selected MPM patients [mPFS of 9.2 months (95% CI, 8.5–10.5)] (7). Furthermore, the results of univariate analysis of outcomes with platinum/pemetrexed did not indicate that BAP1 alterations have any predictive ability in association with this type of treatment. These observations, overall, do not support the assumption regarding the extreme sensitivity of BAP1-mutant MPM to platinum-based chemotherapy. Importantly, Kumar *et al*. did not observe an association between the loss of nuclear BAP1 expression and outcomes with platinum-based chemotherapy in MPM either (54). In another retrospective analysis assessing the predictive value of different genomic aberrations in MPM performed by Lo Iacono et al., PIK3CA and TP53 mutations, but not BAP1 mutations, predicted time-to-tumor progression and OS with platinum/pemetrexed (38). With regards to predictive value of BAP1 aberrations with another chemotherapy regimens, a non-significant trend toward improved OS with vinorelbine in MPM with loss of nuclear BAP1 expression was observed by Kumar *et al*., suggesting a potential modulatory effect of BAP1 on microtubule organization and, as a result, response to vinorelbine (54). In addition, non-functional BAP1 has been associated with resistance to gemcitabine in cell lines (55, 56).

In our series, the presence of BAP1 aberrations did not seem to modify response to ICPi. Indeed, treatment with ICPi, mainly PD-1/PD-L1 inhibitors, resulted in similar ORR and mPFS of 0 and 27% (p-0.28), and 2.5 months (95% CI, 1.4–3.7) and 3.0 months (95% CI, 1.3–10.5) (p-0.39) in BAP1-altered and non-selected MPM patients, respectively. Again, similar ORR (33%, p-0.35) and mPFS [2.0 months (95% CI, 1.9-NR), p-0.96] with ICPi were seen in wild-type BAP1 tumors. The outcomes in both groups were in line with the reported in the literature with PD-1/PD-L1 inhibitors in similar clinical scenario (ORR of 9–29%, mPFS of 2.6–4.1 months) (13–17). Furthermore, univariate analysis of outcomes with ICPi did not demonstrate a correlation between the presence of BAP1 alteration and ICPi efficacy.

The presence of BAP1 aberration in our cohort was associated with numerically longer OS since MPM diagnosis of 98.3 months (95% CI, 9.7–98.3), as compared to 19.4 months (95% CI, 9.7–47.3) in non-selected MPM and 18.8 months (95% CI, 8.5-NR) in wild-type BAP1 MPM—despite the lack of striking inter-group differences in other baseline patient and tumor characteristics. The inter-group differences in proportion of patients receiving platinum/pemetrexed and PARPi are less likely to explain such a big numerical difference in OS. Similar ORR and PFS with platinum/pemetrexed and ICPi, but numerically longer OS since MPM diagnosis in the BAP1-altered group, in our opinion, reflected favorable natural history and indolent character of the disease, and not necessarily better responsiveness to systemic treatments. Giving the higher frequency of family history of malignancy in the BAP1-mutant cohort (24), we can hypothesize, that in some patients in our cohort BAP1 mutation reflected germline abnormalities, which are known to be associated with longer OS (39, 40). Every attempt to elucidate the nature of the BAP1 genomic alterations in our cohort, unfortunately, was unsuccessful. However, considering the low prevalence of germline BAP1 mutations in MPM [1–7% (25–28)], such an explanation for the longer OS in the BAP1-altered group seems less likely.

In our series, none of the four patients with BAP1-altered MPM demonstrated an objective response with PARPi; three patients demonstrated progressive disease at the first radiological response evaluation. This represents an early lack-of-activity signal of PARPi in the BAP1-altered MPM, however, the response evaluation in our series has been done retrospectively, and no central radiological revision has been performed. PARPi are currently being evaluated in two ongoing phase 2 trials: one trial assessing niraparib in BAP1-altered malignant mesothelioma and other DNA damage response-deficient neoplasms (NCT03207347), and another trial evaluating olaparib in separate cohorts of patients with malignant mesothelioma in accordance with the BAP1 status (NCT03531840); no results have been presented so far. One of the promising biological agents in BAP1—altered MPM is enhancer of zeste homolog 2 inhibitor (EZH2i) tazemetostat. This is based on its selective *in vivo* activity in BAP1-mutant MPM (57), and positive results obtained in a phase 2 trial conducted in BAP1-altered MPM (the study met its primary end point demonstrating a disease-control rate of 51% at 12 weeks) (58). The role of histone deacetylase inhibitors (HDACi) in MPM with BAP1 alterations warrants further exploration as well. BAP1 downregulation increases the sensitivity to HDACi in mesothelioma cell lines (59), and therefore, it would be important to see whether BAP1 aberrations modulate response to vorinostat in VANTAGE 014 study (60).

One of the major limitations of our series, in addition to its retrospective nature, small sample size, and lack of central radiological assessment, is the absence of routine molecular profiling for all MPM patients resulting in a significant chance of contamination of one of the comparator groups (group B, representing the “non-selected” MPM) by the BAP1-altered tumors. Whereas the prevalence of BAP1 somatic alterations (including point mutations, deletions, splice alterations, chromosomal alterations, and copy number loss) in MPM ranges between 20 and 64%—depending on the technology used (22, 26, 29–38), most large series implementing next-generation sequencing or Sanger sequencing report on the prevalence of 20–25% (30, 34, 37). Based on the latter estimation and considering performance of genomic testing for some of the patients in the comparator group, the proportion of BAP1-altered tumors in the comparator group (group B) in our cohort is expected to be around 20-25%. Moreover, the estimation of prevalence of BAP1 somatic alterations of 50% results in the proportion of BAP1-altered tumors in the comparator group (group B) of 43%. Overall, the significant chance of contamination of the “non-selected” comparator group by the BAP1-altered tumors represents an additional important limitation of our analysis. This limitation, at least partially, was addressed by the comparative analysis of BAP1-altered and wild-type BAP1 MPM which demonstrated similar outcomes in both groups. Inability to differentiate between the germline and somatic aberrations in the BAP1 gene represents an additional weakness of the analysis. Finally, although patients in the selected cohort were consecutive patients, these appeared to be mainly “good prognosis” by EORTC and CALGB prognostic scoring systems, reflecting the typical patient population treated at tertiary cancer centers. As a result of this selection bias, there is an uncertainty regarding the representability of the selected cohort. Noteworthy, many of the recent clinical trials assessing novel treatment strategies in MPM have selection bias, which misleads the understanding of these novel treatments’ efficacy in the real-world setting.

Since loss of BAP1 nuclear staining correlates with BAP1 loss-of-function mutations with a sensitivity and specificity of 88 and 97% respectively (61), it would be interesting to assess its predictive value on the clinical outcomes of different systemic treatments. Considering the involvement of several oncological centers, and the absence of formal guidelines for IHC BAP1 assessment, a centralized pathological testing was essential for such analysis. Such a centralized pathological testing, however, was not feasible—which represents an additional important limitation of our analysis.

## Conclusions

According to our retrospective analysis, the presence of BAP1 genomic aberration in MPM does not seem to modulate responses to platinum/pemetrexed or ICPi. Numerically longer OS since diagnosis in BAP1-altered MPM has been observed probably reflecting favorable natural history of this disease subset. In four BAP1-altered MPM patients treated with PARPi no responses have been seen. The clinical research to identify effective biological agents in BAP1-mutant MPM is ongoing.

## Data Availability Statement

The raw data supporting the conclusions of this article will be made available by the authors, without undue reservation.

## Ethics Statement

The studies involving human participants were reviewed and approved by 0391-14-RMC. Written informed consent for participation was not required for this study in accordance with the national legislation and the institutional requirements.

## Author Contributions

Conceptualization: ED, DU, and JB. Methodology: ED, DU, and JB. Validation: ED and DU. Formal Analysis: TS. Investigation: ED and DU. Resources: ED, DU, JB, AM, TG, MM, JD, AA, AZ, OR, and NP. Data curation: ED, DU, and TS. Writing-original draft: ED, AM, DU, and JB. Writing-review and editing: ED, DU, JB, AM, TG, MM, JD, AA, AZ, OR, and NP. Visualization: JD, AM, DU, and JB. Supervision: ED and DU. Project administration: ED. Funding acquisition: NA. All authors contributed to the article and approved the submitted version.

## Conflict of Interest

ED reported grants from Boehringer Ingelheim, personal fees for consulting or advisory services from Boehringer Ingelheim, Roche, Astra Zeneca, Pfizer, MSD, BMS, Novartis, Takeda. JB reported grants from MSD, Roche, Boehringer Ingelheim, AstraZeneca and Pfizer, and personal fees for consulting or advisory services from MSD, Roche, Boehringer Ingelheim, AstraZeneca, Pfizer, BMS, Novartis, Takeda, Bayer, Vascular Biogenics, and Abbvie. AM reported honoraria from Merck, Roche. MM reported personal fees for consulting or advisory services from Boehringer Ingelheim, Roche, Astra Zeneca, MSD, BMS, and Takeda. AZ reported grants from BMS, personal fees for consulting or advisory services from Roche, MSD, BMS, Astra Zeneca. NP reported grants and personal fees for consulting or advisory services from Astra Zeneca, Boehringer Ingelheim, BMS, Eli Lilly, MSD, Roche, Pfizer, Novartis, NovellusDx, FMI, Gaurdant360. DU reported personal fees for consulting or advisory services from Boehringer Ingelheim, Roche, Astra Zeneca, MSD, BMS, Takeda.

The remaining authors declare that the research was conducted in the absence of any commercial or financial relationships that could be construed as a potential conflict of interest.

## Abbreviations

AJCC, American Joint Committee on Cancer; BAP1, BRCA1 associated protein-1; CALGB, Cancer and leukemia group B; CI, confidence interval; CT, platinum/pemetrexed+/−antiangiogenic drug; CR, complete response; CTCAE, v. 4.03, Common Terminology Criteria for Adverse Events, version 4.03; CTLA-4, cytotoxic T-lymphocyte-associated protein 4; DNA, deoxyribonucleic acid; ECOG PS, Eastern Cooperative Oncology Group performance status; ed, electronic databases; EORTC, European Organization for Research and Treatment of Cancer; EPP, extra-pleural pneumonectomy; EZH2i, enhancer of zeste homolog 2 inhibitors; HDACi, histone deacetylase inhibitors; HR, hazard ratio; ICC, Israeli cancer centers; ICPi, immune check-point inhibitors; IHC, immunohistochemistry; IQR, interquartile range; MMR, mismatch repair; (m)OS, (median) overall survival; (m)PFS, (median) progression-free survival; MPM, malignant pleural mesothelioma; mRECIST, Modified Revised Response Evaluation Criteria in Solid Tumors; MSI, microsatellite instability; mut/Mb, mutations per megabase; NA, not determined/not available/not applicable; NR, not reached; ORR, objective response rate; OSDx, OS since diagnosis; PARPi, poly (ADP-ribose) polymerase inhibitors; PD, progressive disease; PD-1, programmed cell death-1; PD-L1, programmed cell death ligand-1; PR, partial response; SD, stable disease; SEER, States Surveillance Epidemiology and End Results; TMB, tumor mutational burden; TPS, tumor proportion score.
